# Structural insights into dehydratase substrate selection for the borrelidin and fluvirucin polyketide synthases

**DOI:** 10.1007/s10295-019-02189-z

**Published:** 2019-05-21

**Authors:** Jesus F. Barajas, Ryan P. McAndrew, Mitchell G. Thompson, Tyler W. H. Backman, Bo Pang, Tristan de Rond, Jose H. Pereira, Veronica T. Benites, Héctor García Martín, Edward E. K. Baidoo, Nathan J. Hillson, Paul D. Adams, Jay D. Keasling

**Affiliations:** 1 Department of Energy Agile BioFoundry, Emeryville, CA 94608 USA; 2 Biological Systems and Engineering Division, Lawrence Berkeley National Laboratory, Berkeley, CA 94720 USA; 3 Joint BioEnergy Institute, 5885 Hollis St. 4th Floor, Emeryville, CA 94608 USA; 4 Molecular Biophysics and Integrated Bioimaging Division, Lawrence Berkeley National Laboratory, Berkeley, CA 94720 USA; 5 Department of Bioengineering, University of California, Berkeley, CA 94720 USA; 6 QB3 Institute, University of California, Berkeley, Emeryville, CA 94608 USA; 7 Department of Chemical and Biomolecular Engineering, University of California, Berkeley, Berkeley, CA 94720 USA; 8 Novo Nordisk Foundation Center for Biosustainability, Technical University Denmark, 2970 Horsholm, Denmark; 9 Synthetic Biochemistry Center, Institute for Synthetic Biology, Shenzhen Institutes for Advanced Technologies, Shenzhen, China

**Keywords:** Polyketide, Dehydratase, Borrelidin, Fluvirucin

## Abstract

Engineered polyketide synthases (PKSs) are promising synthetic biology platforms for the production of chemicals with diverse applications. The dehydratase (DH) domain within modular type I PKSs generates an α,β-unsaturated bond in nascent polyketide intermediates through a dehydration reaction. Several crystal structures of DH domains have been solved, providing important structural insights into substrate selection and dehydration. Here, we present two DH domain structures from two chemically diverse PKSs. The first DH domain, isolated from the third module in the borrelidin PKS, is specific towards a *trans*-cyclopentane-carboxylate-containing polyketide substrate. The second DH domain, isolated from the first module in the fluvirucin B_1_ PKS, accepts an amide-containing polyketide intermediate. Sequence-structure analysis of these domains, in addition to previously published DH structures, display many significant similarities and key differences pertaining to substrate selection. The two major differences between BorA DH M3, FluA DH M1 and other DH domains are found in regions of unmodeled residues or residues containing high B-factors. These two regions are located between α3–β11 and β7–α2. From the catalytic Asp located in α3 to a conserved Pro in β11, the residues between them form part of the bottom of the substrate-binding cavity responsible for binding to acyl-ACP intermediates.

**Electronic supplementary material:**

The online version of this article (10.1007/s10295-019-02189-z) contains supplementary material, which is available to authorized users.

## Introduction

Polyketide natural products are one of the largest classes of secondary metabolites, possessing vast structural and chemical diversity. The collinear biosynthetic logic of type I modular polyketide synthases (PKSs) make them a promising synthetic biology platform for the production of existing and novel compounds. The dehydratase (DH) domain within type 1 modular PKSs is responsible for the dehydration of specific C-3 hydroxyacyl-acyl carrier protein (ACP) intermediates, resulting in a corresponding enoyl-ACP [42]. These unsaturated ACP-tethered intermediates can be either reduced by an associated enoyl reductase (ER) domain or result in the production of alkenes in even-to-odd positions in the final polyketide structure [9]. The dehydratase domain from type 1 modular PKSs contains a canonical double hot-dog fold motif with an invariant His/Asp catalytic dyad. These structural features are well conserved and well depicted in DH domain crystal structures from erythromycin [25], curacin [3, 15], rifamycin [17] and the gephyronic acid PKSs [9]. Despite having several crystal structures of DH domains, the structural basis for substrate selectivity and specificity is not completely understood. There is a need to understand substrate selection to enable effective design of synthetic PKSs with different DH substrates. Two main factors important for substrate selection by DH domains include the stereochemistry of the C-3 hydroxyacyl group and the chemical structure of the acyl-ACP intermediate past the C-3 hydroxyacyl position. Dehydration proceeds via a *syn*-coplanar elimination of water and is, therefore, sensitive to the stereochemical configuration of the C-3 hydroxyacyl-ACP substrate [37], which is determined enzymatically by the ketoreductase (KR) domain within the module. Thus, DH domains are tied to KR domains that precede them biochemically [5].

**Fig. 1:**
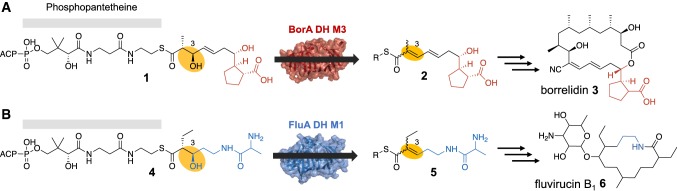
Corresponding substrates (**1**, **4**) and product intermediates (**2**, **5**) for the BorA DH M3 and FluA DH M1. The final polyketide products are illustrated on the right (**3**, **6**). **a** The borrelidin PKS utilizes a *trans*-cyclopentane-dicarboxylate starter unit (highlighted in red) and **b** the fluvirucin B_1_ PKS accepts an amide-containing polyketide starter unit (highlighted in blue). The dehydration reaction is highlighted in yellow. The full biosynthetic polyketide synthase pathways for borrelidin and fluvirucin B_1_ are depicted in Fig. S1 (colour figure online)

The least understood factor in DH substrate selection is the length and chemical structure of the moiety past the C-3 hydroxyl group of the acyl-ACP intermediate. The structure and length of the acyl-ACP intermediate are determined by the upstream PKS module enzymatic architecture and the diverse starter-units incorporated in the nascent polyketide intermediate. How DH domains accommodate these diverse chemical structures while acting on the conserved C-3 hydroxyacyl position of the substrate remains unclear. To further understand substrate selection by DH domains, we conducted a sequence-structure analysis between DH domains from two chemically diverse modular type I PKSs. We chose to investigate two PKSs with distinct starter units and diverse enzymatic architecture: the macrolactone-producing borrelidin (**3**) PKS, and the macrolactam-producing fluvirucin B_1_ (**6**) PKS (Fig. 1, Fig. S1). The borrelidin PKS from *Streptomyces parvulus* Tü4055 utilizes a rare *trans*-cyclopentane-1,2-dicarboxylate starter unit (Fig. 1a, Fig. S1A) [31]. To date, there are very few PKSs identified that are able to incorporate and extend a carboxylate-containing polyketide intermediate. The fluvirucin B_1_ PKS utilizes amide-containing dipeptidyl intermediate **4** (Fig. 1b, Fig. S1B), likely derived from l-aspartic acid and l-alanine [7, 27, 29]. Here, we present the crystal structures of the borrelidin DH domain from module 3 (BorA DH M3) and the fluvirucin B_1_ DH domain from module 1 (FluA DH M1) at 1.80 Å and 2.01 Å, respectively. The BorA DH M3 is specific towards *trans*-cyclopentane-carboxylate-containing polyketide substrate **1**, while the FluA DH M1 accepts amide-containing dipeptide polyketide intermediate **4** (Fig. 1). The DH monomers from both BorA DH M3 and FluA DH M1 possess the traditional double-hotdog fold with an invariant His/Asp catalytic dyad. A close inspection of both BorA DH M3 and FluA DH M1 reveal key structural differences in flexible regions, as observed by high-B-factor analysis, located in the substrate-binding region. In silico docking with their native substrates further supports the importance of these flexible regions. In addition, we aligned DH domain sequences within the ClusterCAD database [13] to identify residues and structural regions that may play a role in substrate binding.

## Results

### Structure analysis

**Fig. 2:**
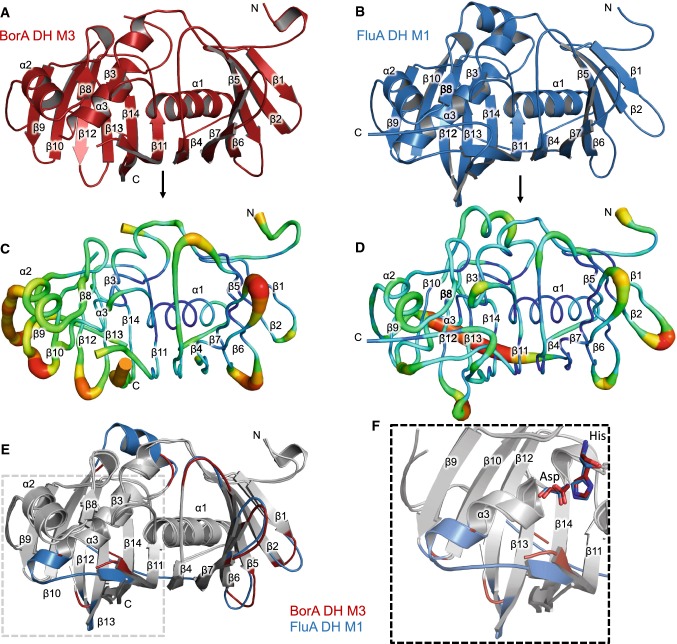
Structure of the BorA DH M3 and FluA DH M1. Overall structure of the BorA DH M3 colored in red **a** and the FluA DH M1 colored in blue **b**. B-factor putty representation of BorA DH M3 **c** and FluA DH M1 **d**, where thin, blue tubes correspond to low B-factors and thicker, darker red tubes correspond to higher B-factors. **e** Structural alignment of the BorA DH M3 and FluA DH M1. Colored regions correspond to regions of higher α-carbon backbone deviation. **f** Close-up of **e** displaying the His/Asp catalytic dyad and the disordered region between α3 and β11, which form part of the substrate-binding cavity (colour figure online)

The BorA DH M3 and FluA DH M1 possess the conventional double-hotdog fold homologous to previously determined type 1 DH domains from the erythromycin, curacin, rifamycin and gephyronic acid PKSs (Fig. 2a, b) [3, 9, 17, 25]. Both BorA DH M3 and FluA DH M1 maintain the canonical His/Asp catalytic dyad (Fig. 2f) and share a sequence identity of 45.0%. Structural alignment between the BorA DH M3 and FluA DH M1 display minimal structural deviation within the double-hotdog motif (Fig. 2e). Cα superposition results in RMSD values of 1.29 Å within 209 residues. As reference with other type I DH domains, both BorA DH M3 and FluA DH M1 share a sequence identity of 45.5% with the erythromycin module 4 DH domain. Cα superposition between the erythromycin module 4 DH domain with either BorA DH M3 and FluA DH M1 results in RMSD values of 1.70 and 1.69 Å over a span of 236 residues. Compared to the dual-functioning dehydratase/isomerase DH domain from the gephyronic acid pathway, both BorA DH M3 and FluA DH M1 share 25.6% sequence identity. Cα superposition between the GphF DH1 and either BorA DH M3 or FluA DH M1 results in higher RMSD values of 2.32 and 2.42 Å over a span of 226 residues. The increase in structural differences between BorA DH M3, FluA DH M1 and GphF DH1 are not surprising, given that GphF DH1 has greater separation within the His/Asp catalytic dyad and other conserved residues surrounding the catalytic region [9].

Most of the structural deviations between BorA DH M3, FluA DH M1 and other DH domains are evident in multiple loop regions connecting the α-helices and β-sheets of the core double-hotdog motif (Fig. 2e). Small loops connecting β1–β2, β5–β6, β6–β7 and β12–β13 displayed small structural deviations. However, the largest deviations were located in two loop regions connecting β7–α2 and α3–β11 (Fig. 2F, Fig. S2). This was further supported by either high B-factors within these residues or missing electron density for residues in the loop region (Fig. 2c, d). In the case of BorA DH M3, we were unable to model 11 residues between α3 and β11, suggesting a highly flexible region within the DH domain (Fig. S2A). A lack of modeled residues between α3 and β11 is also observed in the erythromycin M4, phthiocerol dimycocerosate, curacin F and the rifamycin M10 DH domains (Fig. S2C, E, G, I). Unlike the BorA DH M3, all residues between α3 and β11 were modeled in the FluA DH M1 (Fig. S2B). A closer inspection of the residues between α3 and β11 FluA DH M1 displayed partially higher B-factors. These higher B-factors between α3 and β11 FluA DH M1 are also observed in the curacin H, J, K and gephyronic acid DH domains (Fig. S2D, F, H, J). Overall, B-factor analysis and lack of electron density between α3–β11 and β7–α2 in type I DH structures suggest a highly flexible region within the domain.

### Substrate-binding cavity

**Fig. 3:**
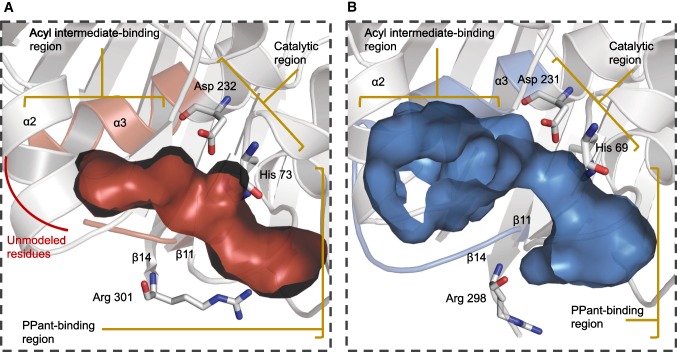
Substrate-binding cavity of the BorA DH M3 and FluA DH M1. **a** Close-up of the BorA DH M3 substrate-binding cavity, key residues and depiction of the three regions. The three binding regions include the PPant-binding, catalytic and acyl intermediate-binding region. Due to poor electron density, 11 amino acids between α3 and β11 are unmodeled, therefore, given an incomplete model of the substrate-binding cavity. **b** Close-up of the FluA DH M1 substrate-binding cavity. The variable loop regions are highlighted in red and blue residues of the cartoon model (colour figure online)

Structural and biochemical data suggest the DH substrate selection is based on DH/ACP protein–protein interactions, stereospecificity of the 3-hydroxyl group and substrate specificity (Fig. S3) [14, 16, 25, 30]. Within each DH monomer, the substrate-binding cavity contains (1) a hydrophobic tunnel/phosphopantetheine (PPant)-binding region, (2) a catalytic region and (3) an acyl intermediate-binding region (Fig. 3). The PPant-binding region is located at the surface of the DH domain between the β11 and β4, in close proximity to β14. An invariant arginine residue on the C-terminal end of β14 has been examined and proposed to be important for either ACP docking and/or PPant recognition [6, 25, 30]. An alignment of BorA DH M3 and FluA DH M1 with other type 1 DH domains displays the conserved arginine residue on the surface of the DH domain (Fig. S4). Measurement between the α-carbon of the conserved Arg and the α-carbon of the catalytic His is on average 17.8 Å (Fig. S4A–B). This highly conserved distance can accommodate the smaller PPant-tethered 3-hydroxyacyl intermediate to the catalytic region. On average, the distance between the phosphate group of the PPant and the C-3 hydroxyl position is 16.4 Å. This is evident in the co-crystal structure of PpsC DH complexed with *trans*-dodec-2-enoyl-CoA (Fig. S4C–E) [14]. These results suggest that the Arg and the residues outlining the substrate tunnel towards the catalytic region in BorA DH M3 and FluA DH M1 are highly conserved amongst type 1 DH domains and may interact extensively with the PPant moiety of the ACP-tethered substrate.

The catalytic region of the DH domain contains the catalytic His and Asp dyad responsible for the dehydration reaction via a *syn*-coplanar elimination of water. In addition to the catalytic His and Asp, both BorA DH M3 and FluA DH M1 contain conserved Leu and Tyr residues that outline the catalytic region, the latter orienting a water molecule (Fig. S5). The catalytic His is located at the beginning of β3 and is on average less than 3.8 Å away from the Asp, located within α3. Recent findings in the fostriecin DH domain suggest DH domains utilize a single-base mechanism, where the active site His residue acts as the base to deprotonate C-2, subsequently protonating the C-3 hydroxyl group to promote C–O bond-cleavage and elimination of water [42]. The carboxylate group of the Asp likely binds and orients the hydroxyl group of the substrate in the favored conformation [42].

The acyl intermediate-binding region can be defined as the residues outlining the bottom of the substrate-binding cavity, outside of the catalytic region. These residues bind to the chemical moiety past the C-3 hydroxyacyl group of acyl-ACP intermediate. The polyketide intermediate past the C-3 hydroxyacyl group can vary in chemical structure, length, and atom heterogeneity, and is dependent on both the upstream PKS module enzymatic architecture and the diverse starter-units incorporated in the nascent polyketide intermediate. The BorA DH M3 can select for a cyclopentane-carboxylate-containing intermediate (Fig. 1a), while the FluA DH M1 can select for an amide-containing dipeptidyl intermediate (Fig. 1b). Both of these substrates require interactions with residues of distinct chemical properties on the acyl-binding region of the DH domain. A thorough inspection of the polyketide acyl-binding region in BorA DH M3 and FluA DH M1 identified residues located between α3–β11 and α2–β8 (Fig. 3). Surprisingly, the same flexible loop region between α3 and β11, where residues are either missing (BorA DH M3) or have higher B-factors (FluA DH M1), form part of the acyl-binding region. Amino acid preference and multiple sequence alignment analysis suggest that most DH domains have a variant loop region between α3 and β11 (Fig. 5b, Fig. S6). Starting from the catalytic Asp in the middle of α3 to a conserved Pro at the beginning of β11, the loop region can vary in length and amino acid composition. These structural insights suggest a putative binding region responsible for selecting the diverse chemical moieties of different polyketide intermediates within DH domains.

### Docking simulations of PPant-tethered substrate intermediates

In our efforts to further validate the importance of the acyl intermediate-binding region for substrate binding and identify key substrate-binding residues, we initially aimed to co-crystalize BorA DH M3 and FluA DH M1 with their corresponding starter units. These starter units included the *trans*-cyclopentane-dicarboxylic acid and β-alanine, in both the free acid and the *N*-acetyl-cysteamine thioester (SNAC)-coupled form. However, no electron density for the corresponding substrates was observed. The lack of substrate binding may be due to high specificity of both BorA DH M3 and FluA DH M1 for their native PPant-tethered polyketide intermediates **1**, **4** (Fig. 1). Using an inactive form of BorA DH M3 and FluA DH M1 with the corresponding native PPant-substrates would greatly increase our efforts in obtaining a substrate–DH domain complex structure. This is supported by the inactive H/F959 PpsC DH domain complexed with a PPant-containing *trans*-dodec-2-enoyl-CoA substrate [14]. Given the complexity to synthesize the native PPant-tethered borrelidin [20] and fluvirucin intermediates for co-crystallization experiments, we decided to conduct in silico docking as an alternative approach. Our initial docking analysis was focused on the crystal structure of FluA DH M1 and its corresponding native substrate **4**. The lack of 11 unmodeled residues in the BorA DH M3 acyl intermediate-binding region would yield inconclusive in silico docking results (Fig. 3a). Therefore, we opted not to conduct substrate docking analysis with the BorA DH M3 model.

**Fig. 4:**
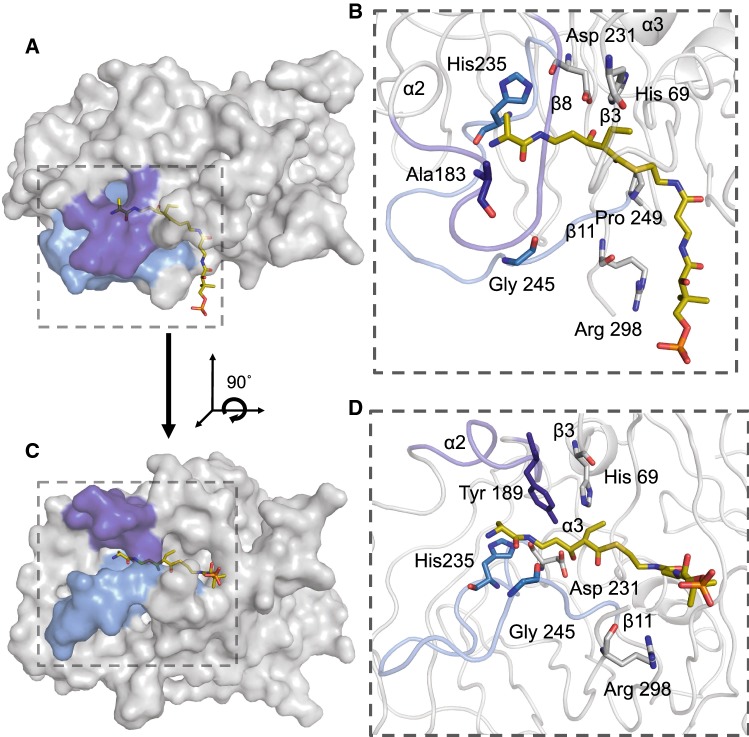
In silico docking analysis of FluA DH M1 with the natural PPant-substrate intermediate **4**. Substrate **4** interacts with all three DH substrate-binding regions **a**. **b** A close inspection of the dipeptide moiety of **4** shows close interactions with residues located between α3 and β11 (light blue) and α2 and β8 (dark blue). **c** A 90˚ horizontal rotation of **a** and close-up of the substrate **4** and interacting residues (colour figure online)

In silico docking of the alanine-protected dipeptide PPant intermediate **4** with the FluA DH M1 revealed key structural regions and residues that may play a role in substrate selection. The fluvirucin PPant-tethered intermediate **4** revealed close interactions with all three regions of the substrate-binding cavity of FluA DH M1 (Fig. 4). On the surface of the DH domain, the conserved Arg 298 was in close proximity for potential electrostatic interactions with the phosphate group of **4**. Several hydrophobic residues (Leu 248, Phe 251, Ala 105) in the PPant-binding region of the DH were in close contact with the PPant moiety of **4**. Within the catalytic region, the C-3 hydroxyl group of **4** is in proximity and oriented toward the catalytic Asp 231. The orientation of the C-3 hydroxyl towards the Asp 231 positioned the C-2 of **4** next to the catalytic His 69, supporting the mechanism of deprotonation of C-2 by the single catalytic base His 69 [42].

Several residues in the acyl intermediate-binding region were in the vicinity of the nitrogen-containing dipeptide moiety of **4** (Fig. 4). The variable loop region between α3 and β11 (light blue) contained polar residues His 235 and Glu 244 within 4.0 Å from the terminal amino group of **4**. In addition, residues between α2 and β8 (dark blue), on the distal side of the variable loop region between α3 and β1, interacted with the dipeptide moiety of **4**. Residues between Ser 185 and the conserved Tyr 189 were in close contact with **4**. Surprisingly, the PPant-tethered fluvirucin intermediate **4** does not accommodate the entire catalytic region of the acyl intermediate-binding region. On average, there is a 9.5-Å distance gap between the terminal amino moiety of **4** and the bottom of the substrate-binding cavity. The bottom of the cavity contains several variant polar residues (Glu 241 and Gln 242). The gap identified between these polar residues with **4**, the high B-factors associated within the invariant loop and the lack of modeled residues for similar type I DH domains suggest this structural region may adopt a distinct conformational change upon binding to the native substrate. This is further supported by the small structural conformational changes observed between the variable loop regions α3–β11 of the *apo* FluA DH M1 crystal structure and the energy minimized docked PPant intermediate **4**-FluA DH M1 complex structure (Fig. S7). Cα superposition results in RMSD values of 2.10 Å within the 18 residues of the α3–β11 loop region.

### Analysis of DH domain conservation

**Fig. 5:**
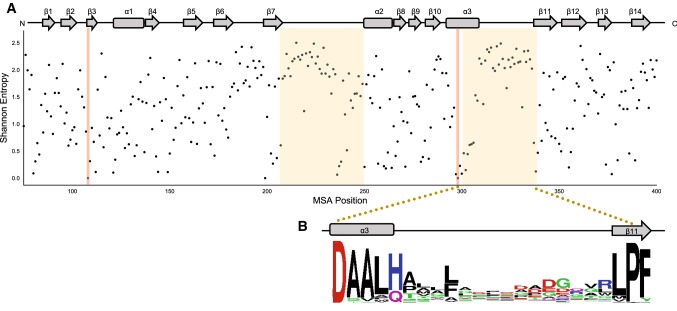
**a** Prediction of conserved residues through Shannon entropy measurements on a multiple sequence alignment of modular type I DH domains taken from ClusterCAD. Low Shannon entropy measurements correspond to low levels of amino acid substitution. The secondary structure of the FluA DH M1 was used to give a relative position within the MSA. Regions highlighted in red display the His/Asp catalytic dyad and the regions highlighted in yellow correspond the two most highly variable regions amongst the known modular type I DH domain structures. **b** Close-up of the region between α3 and β11. The overall height of the stack indicates the sequence conservation at that position (colour figure online)

To quantitatively examine the level of sequence conservation amongst DH domains, the Shannon entropy of aligned DH sequences was calculated at each position within a refined multiple sequence alignment (MSA). 330 DH domain sequences derived from modular type I PKSs were exported from the ClusterCAD database [13]. The secondary structure of the FluA DH M1 was used to refine the DH boundaries of the MSA and visualize Shannon entropy (*H*) at each position (Fig. 5a). Overall, residues that were conserved and displayed low *H* values were both catalytic residues and residues important for structural integrity of the double-hotdog fold motif. The catalytic His/Asp dyad, located within β3 and α3, and related DH motifs (HxxxGxxxxP, GYxYGPxF, LPFxW) displayed low *H* values (Fig. 5a). Similarly, the known structural motifs that make up the DH boundaries (HPLL and LxLxR) prior to β1 and within β14 show low Shannon entropy values.

With the aim of identifying residues and/or structural regions that may play a role in substrate binding past the C-3 hydroxyacyl position, we looked for residues that may be easily substituted with others to accommodate the diversity of the acyl-ACP substrate intermediate. A closer inspection of DH domains showed two regions with high *H* values. These higher *H* values were present in the loop regions between β7–α2 and α3–β11 (Fig. 5b). As observed in the BorA DH M3, FluA DH M1 and other DH structures, these two regions with higher *H* values are located in the same region of unmodeled, high-B-factor residues in the PDB structures. Residues between α3 and β11, and within α2 form part of the acyl intermediate-binding region (Figs. 5, S6). These results further support our DH domain structural and in silico docking analysis regarding the importance of α3–β11 and α2 in substrate selection. These two variant regions between α3–β11 and β7–α2 may possess distinct amino acid properties across different DH domains and may be important for the structural architecture of the acyl intermediate-binding region. In particular, the residues between α3 and β11 and within α2 may play a direct role in substrate selectivity past the C-3 hydroxyacyl position.

## Discussion

Substrate selection by DH domains is attributed to both the stereochemistry of the C-3 hydroxyacyl group and the chemical structure on the acyl-ACP intermediate past the C-3 hydroxyacyl position. The recent structural and biochemical insights of various DH domains have significantly advanced our understanding of stereoselection by DH domains [3, 15, 17, 25, 26]. However, DH substrate selection pertaining to the acyl-ACP intermediate past the C-3 hydroxyacyl position remains elusive. For example, the Rif DH M10, CurK DH, and most DH domains act on a C-3 hydroxyl-ACP intermediate. Yet, the Rif DH M10 from rifamycin is selective towards a C-18, naphthoquinone-containing intermediate [17, 38] while the CurK DH acts on a shorter thiazoline, cyclopropyl-containing intermediate [3, 8]. A key question in DH substrate selectivity is what residue(s) and/or structural features within the DH domain are responsible for selection of the acyl intermediate past the C-3 hydroxyacyl position. Further structural and biochemical insights on DH domains would provide a better understanding of substrate selection past the PPant and C-3 hydroxyacyl moiety.

To further improve our current understanding of DH selectivity towards different acyl-ACP intermediates past the C-3 hydroxyacyl position, we have performed structural analysis on two chemically diverse DH domains. The first DH domain, isolated from the third module in the borrelidin PKS, is specific towards a *trans*-cyclopentane-carboxylate-containing polyketide substrate. The second DH domain, isolated from the first module in fluvirucin B_1_ PKS, accepts an amide-containing polyketide intermediate. Both DH domains maintain the canonical double-hotdog fold motif with the invariant His/Asp catalytic dyad. Surprisingly, a structural comparison of the BorA DH M3, FluA DH M1 and other type I DH domains show few significant deviations from each other. The two major differences between BorA DH M3, FluA DH M1 and other DH domains are found in regions of unmodeled residues or residues containing high B-factors. These two regions are located between α3–β11 and β7–α2. From the catalytic Asp located in α3 to a conserved Pro in β11, the residues between them form part of the bottom of the substrate-binding cavity responsible for binding to the acyl-ACP intermediates past the C-3 hydroxyacyl position. The distal region from α3 to β11 is also part of substrate-binding cavity and is located in α2–β8. The loop connecting β7–α2 is one of the most variable regions in modular type I DH domain structures. The variable β7–α2 loop region may provide flexibility to part of the substrate-binding cavity located in α2–β8 (Fig. 4). These structural insights suggest both α3–β11 and β7–α2 are important for binding of the acyl-ACP intermediate past the C-3 hydroxyacyl position. This was further supported by our in silico docking analysis of the FluA DH M1 and prediction of conserved residues through Shannon entropy measurements (Figs. 4, 5). Structural evidence for this can be observed in the PpsC DH structures in complex with *trans*-dodec-2-enoyl-CoA (PDB ID: 5NJI) or crotonyl-CoA ((PDB ID: 5I0K). Comparison of the α3–β11 and β7–α2 regions in both structures displays key differences. More residues were modeled in the α3–β11 of PpsC DH structure in the presence of the longer *trans*-dodec-2-enoyl-CoA substrate, suggesting higher stability of the α3–β11 region upon binding of a substrate that closely resembles the native acyl-ACP intermediate.

The identification of both the α3–β11 and β7–α2 regions involved in substrate selection may provide insights into rational engineering of DH domains. In our efforts to improve DH-mediated dehydration in adipic acid production using the engineered BorA2 PKS [19], we tested various DH domains and chimeric DH domains with α3–β11 loop swaps in vitro (Fig. S8a). Surprisingly, all chimeric DH domains containing the α3–β11 loop swaps were soluble (Fig. S8b). This suggests that a key secondary structure is maintained within the DH domain’s α3–β11 loop swap region. However, a close inspection of adipic acid production using the chimeric DH domains was inconclusive (Fig. S8c). We speculate that the lack of conclusive results may be due to both the high background of adipic acid that is likely derived from *E. coli* during protein purification, and low efficiency of the BorA2 PKS to produce adipic acid. This assay may not be optimal for testing chimeric DH activity. Nonetheless, generating soluble and stable chimeric DH domains is a first step towards DH engineering. Further efforts in investigating the activity of chimeric DH domains can be simplified by testing standalone chimeric DH domains containing the α3–β11 loop swaps with more naturally relevant acyl-ACP, acyl-PPant or acyl-SNAC substrates [20].

Our structures of the BorA DH M3 and FluA DH M1 provide further structural evidence of substrate selection by DH domains within modular type I PKSs. We present both a sequence-structural analysis between DH domains and identify significant similarities and key differences pertaining to substrate selection. The work presented here in combination with existing DH domain studies should facilitate further engineering efforts of modified PKSs that can process non-natural substrates with improved catalytic properties.

## Materials and methods

### Cloning of *BorA DH M3* and *FluA DH M1*

Vector backbone template originated from JPUB_008800 plasmid (Table S1). The genes encoding for BorA DH M3 and FluA DH M1 were PCR amplified from the pDVA00936 and pTL-A01 plasmids, and cloned into a pET28a vector using Gibson assembly methodology [18]. The j5 DNA assembly design automation software [22] was utilized to generate Gibson assembly primers for the BorA DH M3, FluA DH M1 and pET28a vector backbone. Primer sequences are available in Table S1. Polymerase chain reaction amplification of the BorA DH M3, FluA DH M1 and pET28a backbone was conducted using Phusion Hot Start II DNA polymerase (Thermo Fisher) using the vendor’s recommended protocol at an annealing temperature of 65 °C and 1 min extension time for the DHs and 3 min extension time for the pET28 backbone. The pET28a PCR product was Dpn1 digested (Thermo Fisher) using the vendor-recommended protocol and subjected to PCR clean up (Zymo Research). The DH genes and pET28a PCR product were ligated using Gibson assembly master mix (New England BioLabs), following the vendor-recommended protocol. Both DH constructs were designed with a thrombin-cleavable N-terminal 6 × His-tag. The strains and plasmid sequences utilized in this study are listed in Table S1. All strains and plasmid sequences may be accessed and requested through the Joint BioEnergy’s public registry (https://public-registry.jbei.org/folders/412) [21].

### Protein expression and purification

The recombinant BorA DH M3 and FluA DH M1 containing an N-terminal 6 × His-tag were produced in BL21 (DE3) *E. coli* cells (Novagen). Cells containing the pET28-DH plasmids were grown to OD_600_ = 0.8 at 37 °C in TB medium containing 50 µg/mL kanamycin. The cell cultures were cooled to 18 °C and expression was induced using 1 mM IPTG. The cell cultures were incubated for an additional 16 h at 18 °C and harvested by centrifugation at 5525 r.c.f. for 10 min. The cell pellets were resuspended in 50 mM Hepes, pH 7.6, 10% glycerol, 10 mM imidazole, 300 mM NaCl. Resuspended cells were cooled on ice for 30 min and the cells were disrupted using sonication. The cell debris was cleared by centrifugation at 21,036 r.c.f. for 1 h. The supernatant was collected and batch bound to HisPur TM Cobalt Resin (Thermo Scientific) for 1 h at 4 °C. BorA DH M3 and FluA DH M1 were purified according to the manufacturer’s instructions using an imidazole step-gradient. Fractions containing pure protein were determined by SDS-PAGE and fractions containing either BorA DH M3 or FluA DH M1. Removal of the N-terminal 6 × His-tag was conducted by incubating the fractions containing the proteins at 14 °C for 30 h with thrombin from bovine plasma (Sigma-Aldrich) at a concentration of 2 U/mg of recombinant protein and 3.5 mM CaCl_2_. After thrombin digestion and assessment of N-terminal 6 × His-tag cleavage via SDS-PAGE, we dialyzed the DH domains against 50 mM Hepes, pH 7.6, 5% glycerol. Removal of thrombin and further purification of BorA DH M3 and FluA DH M1 were conducted by anion-exchange chromatography using HiTrap Q FF (GE Healthcare) according to the manufacturer’s instructions. Purified BorA DH M3 and FluA DH M1 were dialyzed against crystallization buffer, which consisted of 25 mM Hepes, pH 7.6, and 1 mM dithiothreitol (Fig. S9). Both BorA DH M3 and FluA DH M1 were concentrated to 12 mg/mL for crystallographic studies.

### Crystallization and structure determination

Crystallization screening was carried out on a Phoenix robot (Art Robbins Instruments, Sunnyvale, CA) using a sparse matrix screening method [23]. BorA DH M3 concentrated to 10 mg/mL was crystallized by sitting drop vapor diffusion in drops containing a 1:1 ratio of protein solution and 0.20 M MgCl_2_, 0.10 M Tris, pH 8.5, and 29% (w/v) PEG 4000. For FluA DH M1, 10 mg/mL protein was crystallized by sitting drop vapor diffusion in drops containing a 1.5:1 ratio of protein solution and 0.40 M NaCl, 0.10 M Tris, pH 8.5, and 29% (w/v) PEG 3350. For both proteins, a final concentration of 15% glycerol was added before flash freezing in liquid nitrogen.

The X-ray data sets for BorA DH M3 and FluA DH M1 were collected at the Berkeley Center for Structural Biology on beamlines 8.2.1 and 8.2.2 of the Advanced Light Source at Lawrence Berkeley National Laboratory. Diffraction data were recorded using ADSC Q315R detectors (Area Detector Systems Corporation, San Diego, CA). Processing of image data was performed using the HKL2000 suite of programs [32]. For both proteins, phases were calculated by molecular replacement with the program Phaser [28], using the structure of Rif DH M10 (PDB id: 4LN9) [6] as a search model. Automated model building was conducted using AutoBuild [39–41] from the Phenix [1] suite of programs resulting in a model that was mostly complete. Manual building using Coot [12] was alternated with reciprocal space refinement using Phenix [2]. Waters were automatically placed using Phenix and manually added or deleted with Coot according to peak height (3.0*σ* in the *Fo*-*Fc* map) and distance from a potential hydrogen bonding partner (< 3.5 Å). TLS refinement [33] using ten groups, chosen using the TLSMD web server [33], was used in later rounds of refinement. All data collection, phasing, and refinement statistics are summarized in Table S2.

### In silico substrate docking

We conducted in silico substrate docking between **4** and the FluA DH M1. The natural PPant-tethered substrate **4** was initially drawn in Chemdraw (PerkinElmer) and transferred in SMILES format to the PHENIX software suite, eLBOW ligand and constraints generator [1]. The eLBOW program generated a PDB file of the PPant-tethered substrate **4** and a constraints (.cif) file. Both files were used to dock **4** into the FluA DH M1. As a reference for substrate binding, we used the co-crystal structure of PpsC DH complexed with *trans*-dodec-2-enoyl-CoA (PDB ID: 5NJI) to position substrate **4** into the FluA DH M1. We utilized the program *Coot* to overlay the PpsC DH with chain A of the FluA DH M1 and model in substrate **4** [11]. The entire FluA DH M1-substrate **4** complex was then energy minimized using the program UCSF CHIMERA [34]. The lack of 11 unmodeled residues in the BorA DH M3 acyl intermediate-binding region made it difficult to conduct precise in silico docking on this target and obtain conclusive information. Therefore, we opted not to conduct substrate docking analysis with the BorA DH M3 model.

### Protein structure and visualization

All of the protein structure analysis and figures were generated using UCSF CHIMERA [34] and PyMOL (The PyMOL Molecular Graphics System, PyMOL1.2edu1 2009, Schrödinger, LLC). Both PyMOL and the online I-TASSER server were used for PDB structural alignment analysis [36]. Multi-sequence alignment for Fig. S6 was generated using MUSCLE [10] and visualized using ESPript 3.0 [35].

### Analysis of DH sequence conservation

The Shannon entropy of aligned DH sequences was calculated at each position within a refined multiple sequence alignment (MSA). Briefly, 330 sequences derived from PKS non-loading modules containing AT, DH, KR, and ACP domains with active KR and DH domains were exported from the ClusterCAD database [13]. Sequences contained the first residue after the AT domain to the last residue immediately preceding the downstream domain (usually a KR or ER). Sequences were then aligned using MAFFT [24]. The MSA was then refined using the ProDy python library so that all positions within the MSA maintained at least 10% occupancy [4]. ProDy was then used to calculate the Shannon entropy of each position within the resulting MSA. The boundaries of the DH domain within the MSA were further refined using PDB structural information.

## Electronic supplementary material

Below is the link to the electronic supplementary material.

Supplementary material 1 (PDF 1776 kb)

## Abbreviations

PK: Polyketide
PKS: Polyketide synthase
Flu: Fluvirucin
Bor: Borrelidin
DH: Dehydratase
ACP: Acyl carrier protein
PPant: Phosphopantetheine
MSA: Multiple sequence alignment
SNAC: *N*-Acetyl-cysteamine thioester
