# Supplementary material for: Structural insights into dehydratase substrate selection for the borrelidin and fluvirucin polyketide synthases
Source: J Ind Microbiol Biotechnol. 2019 May 21;46(8):1225–35. doi: 10.1007/s10295-019-02189-z (PMC6697708; doi:10.1007/s10295-019-02189-z)
Supplement: Supplementary file 1 — Supplementary material 1 (PDF 1776 kb) [file 10295_2019_2189_MOESM1_ESM.pdf]

Supporting information for

Structural insights into dehydratase substrate selection for the borrelidin and fluvirucin polyketide synthases

Jesus F. Barajas [#1,2], Ryan P. McAndrew [#3,4], Mitchell G. Thompson [2,3], Tyler W. H. Backman [2,3,5,6], Bo Pang [2,3,6], Tristan de Rond [2,3], Jose H. Pereira [3,4], Veronica T. Benites [1,2,3], Héctor García Martín [1,2,3], Edward E. K. Baidoo [1,2,3], Nathan J. Hillson [1,2,3], Paul D. Adams [2,3,4,5], Jay D. Keasling [2,3,5,6,7,8,9 *]

[1] Department of Energy Agile BioFoundry, Emeryville, CA 94608, USA
[2] Biological Systems and Engineering Division, Lawrence Berkeley National Laboratory, Berkeley, CA 94720, USA
[3] Joint BioEnergy Institute, Emeryville, CA 94608, USA
[4] Molecular Biophysics and Integrated Bioimaging Division, Lawrence Berkeley National Laboratory, Berkeley, CA 94720, USA
[5] Department of Bioengineering, University of California, Berkeley, CA 94720, USA
[6] QB3 Institute, University of California, Berkeley, Emeryville, CA 94608, USA
[7] Department of Chemical & Biomolecular Engineering, University of California, Berkeley, Berkeley, CA 94720, USA
[8] Novo Nordisk Foundation Center for Biosustainability, Technical University Denmark, DK2970-Horsholm, Denmark
[9] Synthetic Biochemistry Center, Institute for Synthetic Biology, Shenzhen Institutes for Advanced Technologies, Shenzhen, China.

* Correspondence: Dr. Jay D. Keasling, Joint BioEnergy Institute, 5885 Hollis St. 4th floor, Emeryville, CA 94608, USA; Phone: 510-486-2630, E-mail: jdkeasling@lbl.gov

[#] Equal contribution.

Supplementary discussion

Generating and testing chimeric dehydratase domains for adipic acid production

The identification of both the α3-β11 and β7-α2 regions involved in substrate selection provide insights into rational engineering of DH domains. We initially hypothesized that the α3-β11 loop region could be directly involved in substrate selection. To test this hypothesis, we initially designed an *in vitro* experiment where we generated excised chimeric DH domains, swapping out the α3-β11 loop region, and measuring both substrates (**1**, **4**) and products (**2**, **5**) of the native BorA DH M3 and FluA DH M1 (Fig.1) via liquid chromatography-mass spectrometry (LC-MS). The boundaries from the loop swaps began from the catalytic Asp located in α3 to a conserved Pro in β11 (Fig. 5B). However, given the difficulty to synthesize (**1**, **2**, **4**, **5**), we explored alternative biochemical assays to test our hypothesis.

Previously, Hagen and coworkers developed and engineered a polyketide synthase platform from the borrelidin PKS to produce adipic acid *in vitro* [3]. This platform required the integration of a full reductive loop containing a ketoreductase (KR), dehydratase (DH) and enoylreductase (ER). In addition, the engineered PKS platform contained a terminal thioesterase (TE) for the hydrolysis of the adipyl-ACP intermediate to generate adipic acid as the final product (Fig. S8a). In this study, enzymatic dehydration by the BorA DH M2 was one of the main bottlenecks in adipic acid biosynthesis. Using this *in vitro* platform, we tested our chimeric DH domains. This platform did not require the synthesis of complex substrates or products and took advantage of the already established PKS platform to readily test enzymatic dehydration in adipic acid production. We generated several chimeric DH constructs in which we swapped the α3-β11 loop region within the DH domain of adipic acid-producing WT BorA 2 PKS with that of the BorA M3 DH, FluA DH M1 or the erythromycin DH domain (Table S1, Fig. S8). In addition, we completely swapped the BorA M2 DH domain with that of the BorA M3 DH, FluA DH M1 or the erythromycin DH domain (Table S1, Fig. S7). DH chimeric variants were synthesized as gblocks from IDT. Gblocks of variant DH domains contained 40 base pair homology flanking regions to the WT BorA 2 PKS. The WT BorA 2 PKS was amplified containing the 40 base pair homology regions. Gblocks of DH variants and linear BorA 2 PKS were assembled using Gibson DNA assembly, as described in the materials and methods section. Complete sequences of the DH chimera variants, gblocks and primers are available in Table S1.

Purification of PKS variants was conducted as described in *Hagen et al* [3]. Surprisingly, all chimeric DH domains containing the α3-β11 loop swaps were soluble (Fig. S8b). However, some DH chimera variants were less soluble than others (Fig. S8b). This suggests that a key secondary structure is maintained within the DH domain's α3-β11 loop swap region. Intermediate and product analysis was conducted as described in *Hagen et al* [3]. Initially, we tested both succinyl-SNAC and succinyl-CoA as starter units in our *in vitro* reactions. Higher adipic acid production was observed when utilizing succinyl-CoA. Therefore, we conducted all of our testing of DH chimera variants with succinyl-CoA. Each DH chimera variant was tested in three separate experiments in technical triplicates. A close inspection of adipic acid production using the chimeric DH domains was inconclusive (Fig. S8c). We hypothesized that several DH chimera variants, having the BorA M2 DH loop, would be active or partially active and generate

adipic acid as the final product, relative to the WT BorA 2 PKS. However, LC-MS/MS results varied in each experiment. Most DH chimera variants generated adipic acid levels close to the negative control variant, where we inactivated the catalytic histidine of the DH domain in BorA 2 PKS. We speculate that the lack of conclusive results may be due to both the high background of adipic acid that is likely derived from *E. coli* during protein purification, and low efficiency of the BorA2 PKS to produce adipic acid. This assay may not be optimal for testing chimeric DH activity. Further efforts in investigating the activity of chimeric DH domains can be simplified by testing standalone chimeric DH domains containing the α3-β11 loop swaps with more naturally relevant acyl-ACP, acyl-PPant or acyl-SNAC substrates. Moreover, a different *in vitro* PKS platform, such as the already established erythromycin PKS, can be utilized to test DH chimera variants.

Supplementary tables and figures

Table S1. Plasmids and primers used in this study. All strains, plasmid sequences for DH domains used in the structural characterization may be accessed and requested through the Joint BioEnergy's public registry (https://public-registry.jbei.org/folders/412)[4]. Sequences of all the DH chimera variants tested described in the supplementary discussion and displayed in Fig. S8 can also be accessed in (https://public-registry.jbei.org/folders/412)[4].

Strain part ID	Plasmid part ID	Construct summary
JPUB_011380	JPUB_011381	pET28a-BorA_DH_M3
JPUB_011382	JPUB_011383	pET28a-FluA_DH_M1
JPUB_008799	JPUB_008800	pET28a-CaiC
Lin TY *et al*.[5]	Lin TY *et al*.[5]	pTL-A01
JPUB_011378	JPUB_011379	pDVA00936
**Primer**	**Sequence 5'-3'**	
pET28a-Forward	catatggctgccgcgcggcac	
pET28a-Reverse	taaggatccaaactcgagcaccaccacc	
BorA_DH_M3_Forward	gcctggtgccgcgcggcagccatatgcaccccgcaggtgacgt	
BorA_DH_M3_Reverse	gctcgagtttggatcctcatgcctgacggcgtgca	

FluA_DH_M1_Forward	gcgcggcagccatatggcgggggcgtcggacgcgggcg
FluA_DH_M1_Reverse	ctcgagtttggatccttacccagaccgcggacgcgacgac
**Plasmid part ID**	**Construct summary**
JPUB_013570	WT pBK040
JPUB_013552	pPK040 DH Knockout
JPUB_013558	pBK040 BorA DH M2 with FluA DH M1 loop
JPUB_013554	pBK040 BorA DH M3 WT
JPUB_013562	pBK040 BorA DH M3 with BorA DH M2 loop
JPUB_013560	pBK040 BorA DH M3 with FluA DH M1 loop
JPUB_013556	pBK040 FluA DH M1 WT
JPUB_013564	pBK040 FluA DH M1 with BorA DH M2 loop
JPUB_013568	pBK040 DEBS DH WT
JPUB_013566	pBK040 DEBS DH with BorA DH M2 loop
**Primer**	**Sequence 5'-3'**
pBK040_BorA2_PKS_F	ctggttctgcgcagtgccgccgcgcgtcgtacgggtgcccgtcgccag
pBK040_BorA2_PKS_R	atgacgggcgtcgccgagacctaattcggccggttcggcggccgagc

Table S2. X-ray data collection and refinement statistics.

	BorA DH M3	FluA DH M1
PDB ID	6OBT	6OBV
Resolution (Å)	30.9 – 1.80<br>(1.83 – 1.80)	50 – 2.01<br>(2.04 – 2.01)
Space group	C121	C121
Unit cell (Å)	a = 108.2<br>b = 63.4<br>c = 40.7	a = 152.8<br>b = 38.8<br>c = 197.0
	α = 90.0°<br>β = 100.4°<br>γ = 90.0°	α = 90.0°<br>β = 104.0°<br>γ = 90.0°
Total reflections	90869	277031
Unique reflections	24737	75387
Average Multiplicity	1.9 (1.9)	2.0 (2.1)
Completeness (%)	96.2 (94.9)	89.2 (83.6)
<I/σ(I)>	9.7 (1.0)	12.9 (1.2)
CC1/2	0.997 (0.555)	0.999 (0.562)
*R*merge*	0.062 (1.598)	0.080 (1.158)
*R*factor	0.20	0.20
*R*free	0.23	0.26
Wilson B	33.2	30.7
Geometry Statistics		

R.m.s deviations from ideal geometry†		
Bonds (Å)	0.006	0.007
Angles (°)	0.881	0.902
Molprobity Analysis‡		
Ramachandran plot		
Favored (%)	97	97
Outliers (%)	0	0.2
Rotamer Outliers (%)	0	0
Clashscore	4.5	4.3

Statistics for the highest-resolution shell are shown in parentheses. *R_{merge} = S_{hkl} S_i $|I_i(hkl) – (I(hkl))|$/ S_{hkl} S_i $I_i(hkl)$, where <*I*(*hkl*)> is the mean intensity after rejection. † With respect to Engh and Huber parameters[2]. ‡[1].

Figure S1. The borrelidin (A) and fluvirucin B_1 (B) biosynthetic pathways. The DH domain structures presented in this study are highlighted in red and blue.

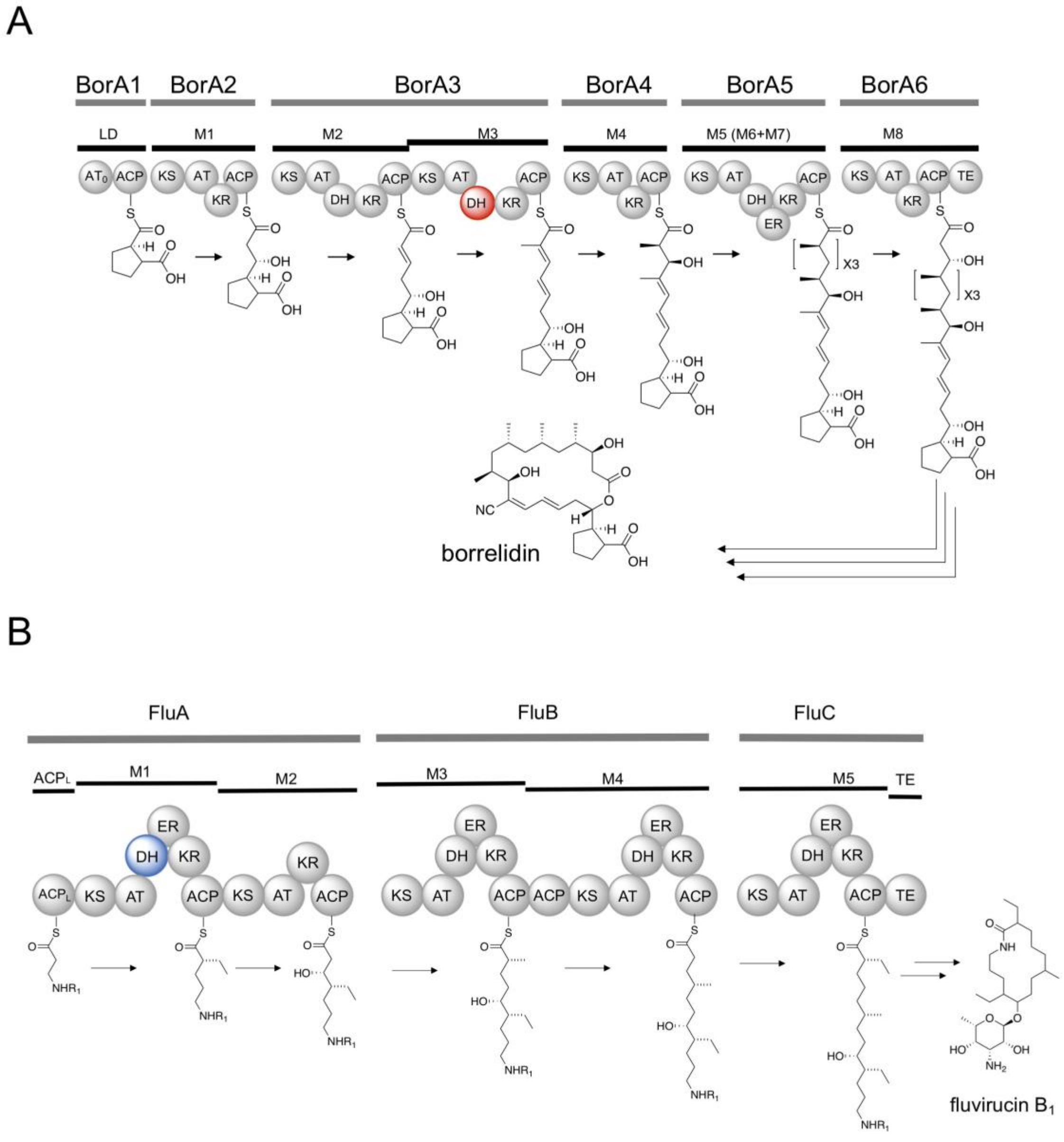

Figure S2. Flexible regions within modular type I DH domains. B-factor analysis between α3 and β11of type I DH domains. Blue thin tubes correspond to low B-factors and thicker, darker red tubes correspond to higher B-factors. In the case of BorA DH M3 (A), were unable to model residues between α3-β11. A lack of modeled residues between α3-β11 is further observed in the erythromycin M4 (C), phthiocerol dimycocerosate (E), curacin F (G), and the rifamycin M10 (I) DH domains. All residues between α3-β11 were modeled in the FluA DH M1 (B). A closer inspection of the residues between α3-β11 FluA DH M1 displayed higher B-factors. These higher B-factors between α3-β11 FluA DH M1 are also observed in the curacin H, J, K (D,F,H) and gephyronic acid (J) DH domains. PDB entries for the DH domains are 6OBT, 6OBV, 3EL6, 3KG7, 5NJI, 3KG8, 3KG6, 3KG9, 4LN9, 6MBG. K) Structural alignment of the previously mentioned DH domains, highlighting the variable loop region between β7-α3.

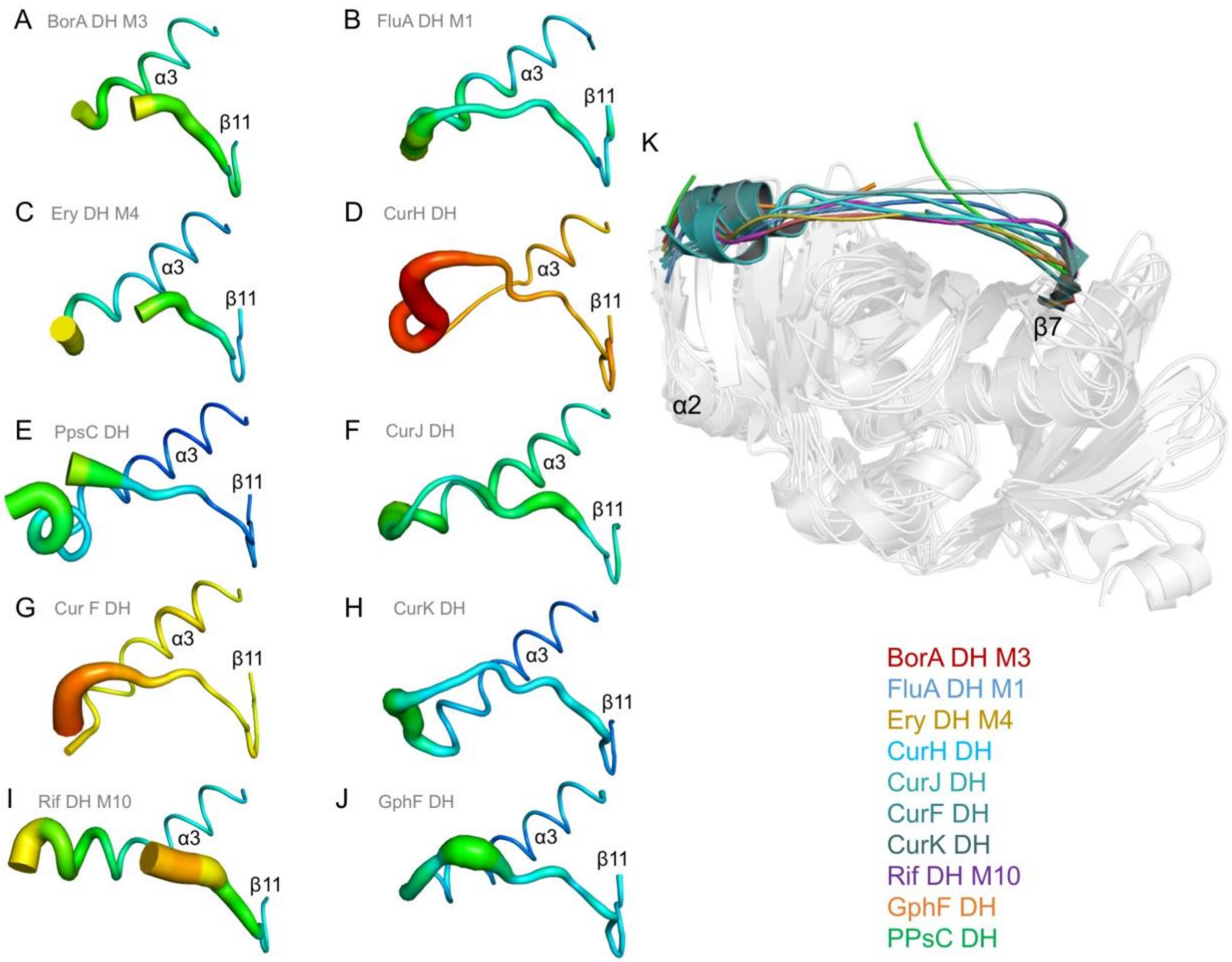

Figure S3. Examples of chemical structures of substrates of type 1 modular DH domains used in our structural analysis. Highlighted in yellow is the C-3 hydroxyacyl position where DH domains catalyze the dehydration reaction. Highlighted in blue and red are the starter unit(s) in fluvirucin and borrelidin biosynthesis.

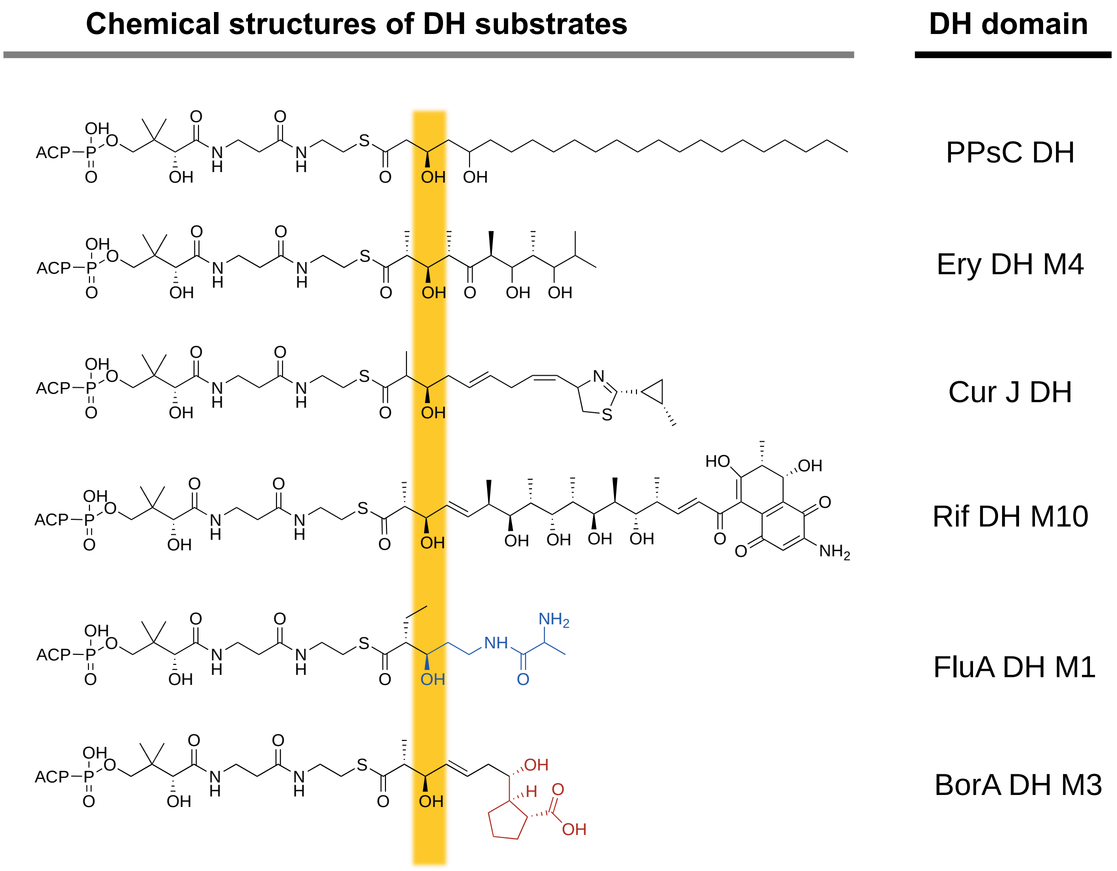

Figure S4. Structural alignment of various type I PKS DH domains highlighting the ACP/ PPant-binding and catalytic residues. (A) Measurement between conserved arginine residues and the catalytic histidine is on average 17.8 Å. (B) A table listing all of the DH domains aligned, including the residues and distance between the α-carbon of the arginine and histidine residues. (C) Structural alignment depicted in (A) with the *trans*-dodec-2-enoyl-CoA substrate co-crystallized in the PpsC DH domain (PDB ID: 5NJI). The catalytic pocket of the PpsC DH domain is displayed in the surface representation around the *trans*-dodec-2-enoyl-CoA substrate. (D) The distance between the phosphate of the *trans*-dodec-2-enoyl-CoA substrate and the C-3 hydroxyacyl position, where DH domains are catalytically active (yellow circle), is on average 16.4 Å. The *trans*-dodec-2-enoyl-CoA substrate closely mimics the native phosphopantetheine arm (E) of the ACP.

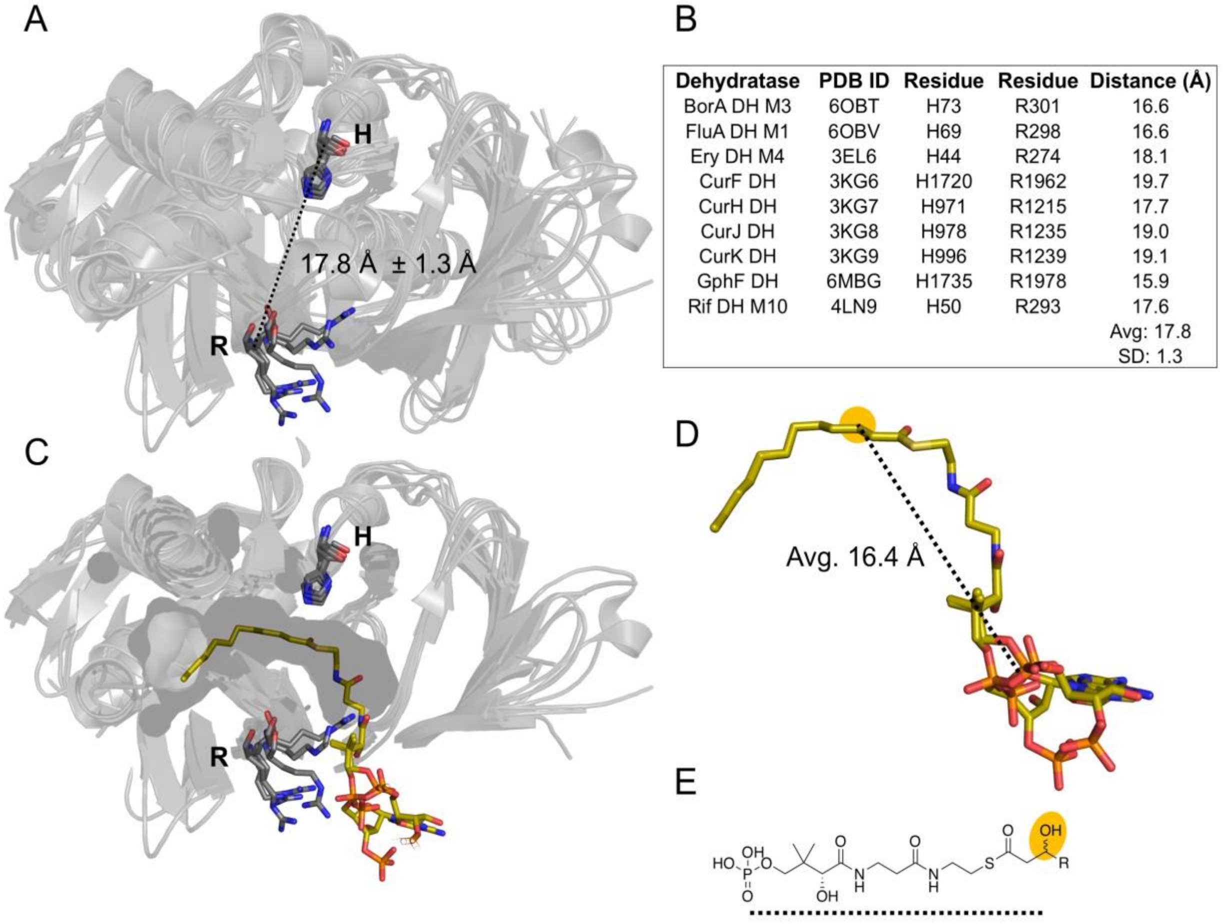

Dehydratase	PDB ID	Residue	Residue	Distance (Å)
BorA DH M3	6OBT	H73	R301	16.6
FluA DH M1	6OBV	H69	R298	16.6
Ery DH M4	3EL6	H44	R274	18.1
CurF DH	3KG6	H1720	R1962	19.7
CurH DH	3KG7	H971	R1215	17.7
CurJ DH	3KG8	H978	R1235	19.0
CurK DH	3KG9	H996	R1239	19.1
GphF DH	6MBG	H1735	R1978	15.9
Rif DH M10	4LN9	H50	R293	17.6
				Avg: 17.8
				SD: 1.3

Figure S5. 2Fo − Fc electron density maps of BorA DH M3 (A) and FluA DH M1 (B) active site. Maps were contoured to 1 σ at 1.80 Å and 2.01 Å respectively.

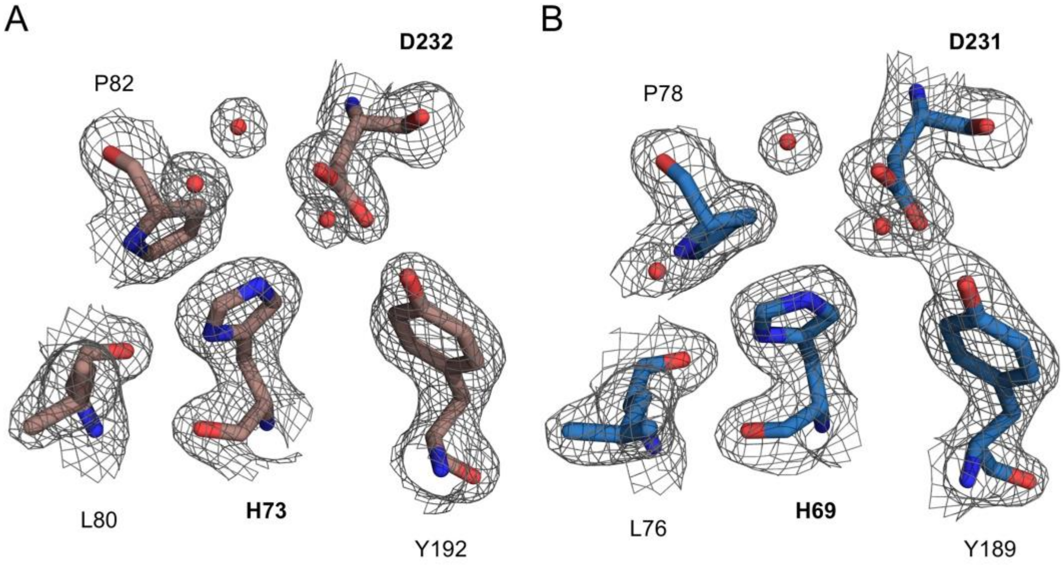

Figure S6. Multiple sequence alignment of BorA DH M3, FluA DH M1 and other previously solved type I DH domains structures.

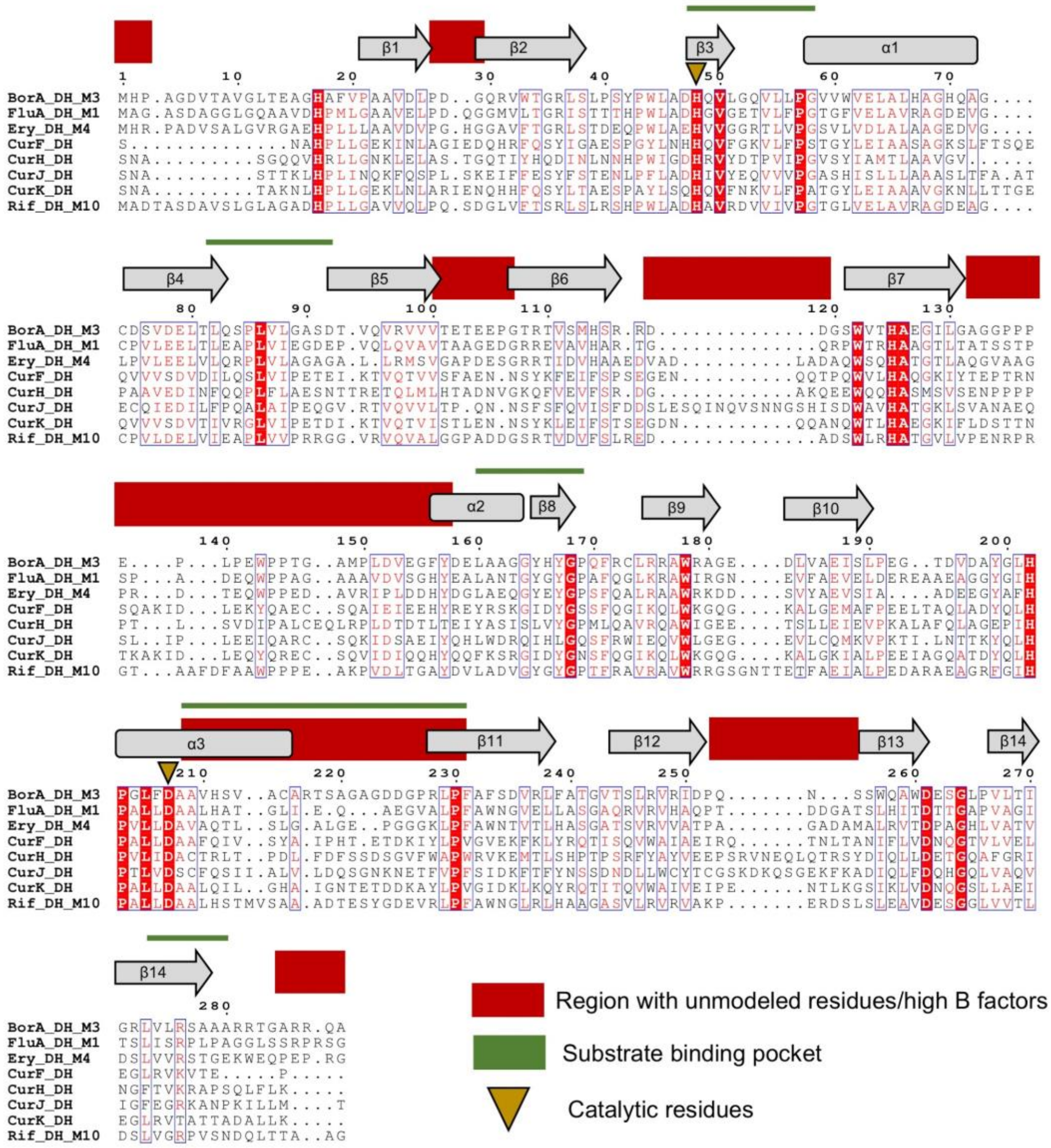

Figure S7. Structural alignment between the *apo* FluA DH M1 and the *in silico* docked-**4**, energy minimized FluA DH M1 (A). Cα superposition results in RMSD values of 2.10 Å within the 18 residues of the α3-β11 loop region (B).

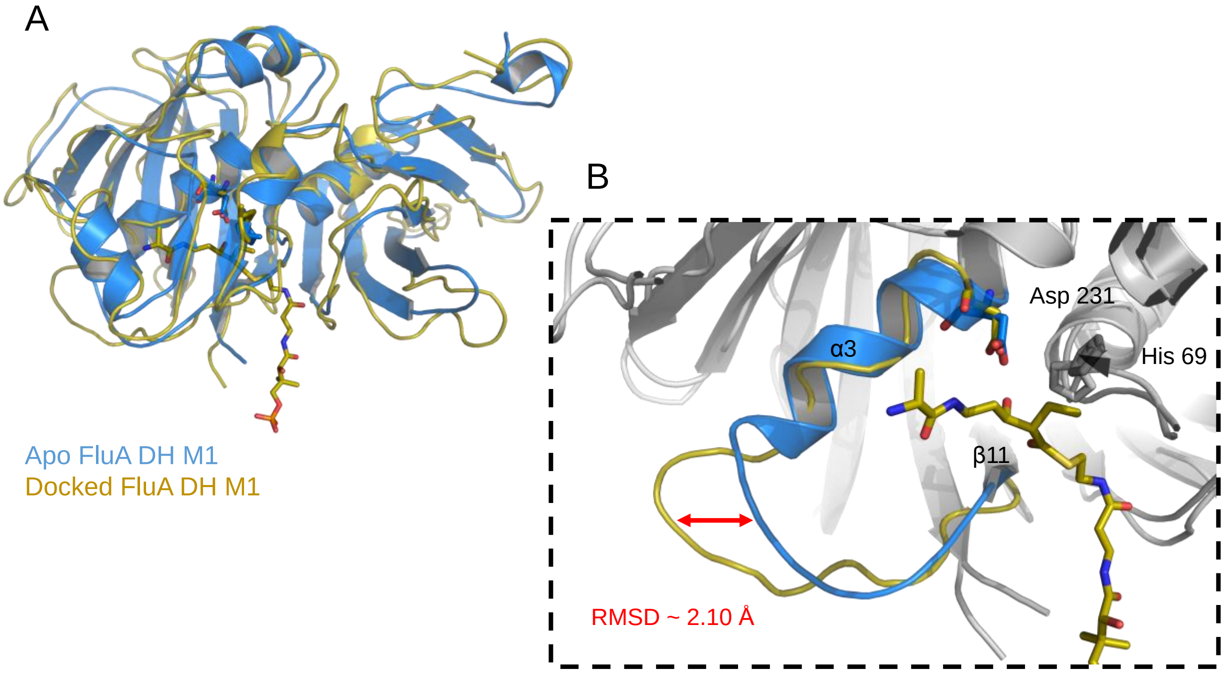

Figure S8. Initial rational DH engineering efforts. To improve DH-mediated dehydration in adipic acid production using the engineered BorA2 PKS[3], we tested various DH domains and chimeric DH domains with α3-β11 loop swaps *in vitro* (A). Surprisingly, all chimeric DH domains containing the α3-β11 loop swaps were soluble (B). This suggest that the overall protein is likely not misfolded and stable. However, a close inspection of adipic acid production of the chimeric DH domains using LC-MS was inconclusive (C). DH chimeras expected to be active or partially active are displayed with a green circle while chimeras expected to have lower or no activity are depicted in red circles. Methods for *in vitro* acid production using BorA2 and LC-MS analysis can be found in Hagen *et al.* 2016 [3].

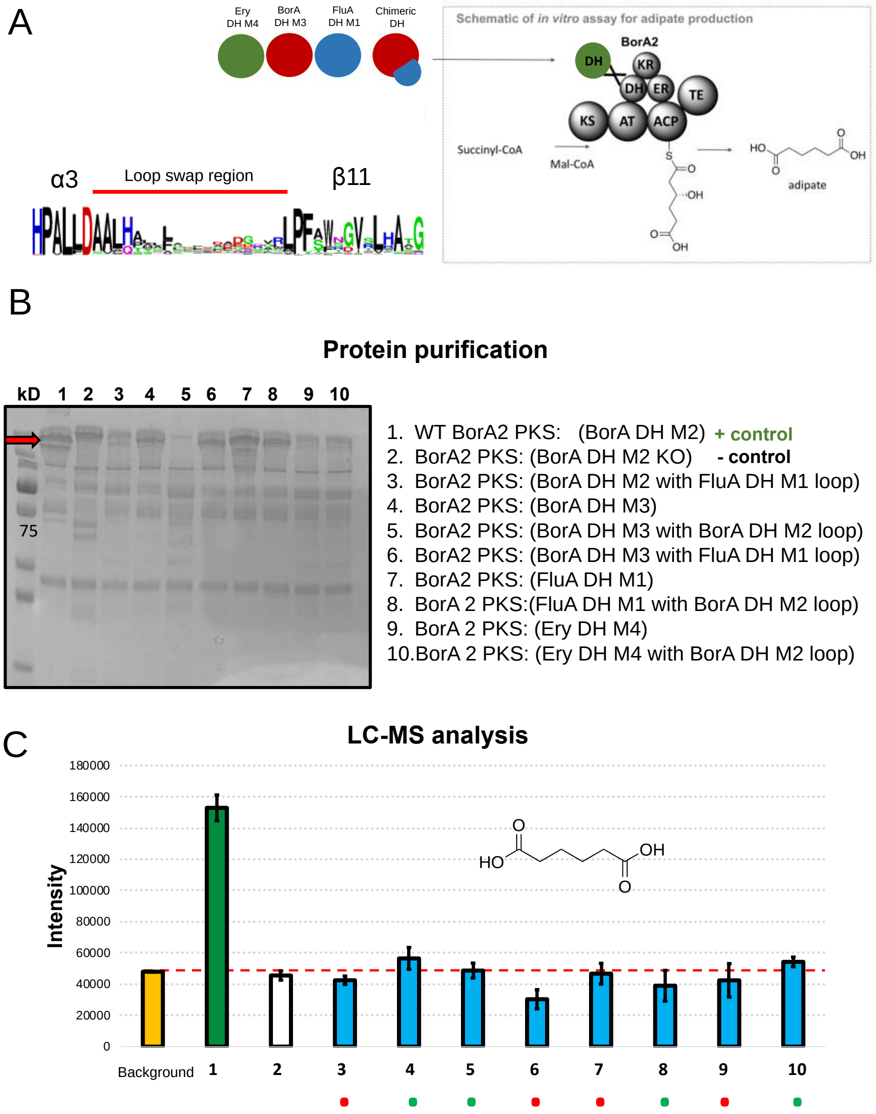

Figure S9. SDS-PAGE of pET28-N-6xHis purified proteins in this study. A Tris-Glycine 8-16% precast gel (Bio-Rad) was used. For molecular weight determination, PageRuler Plus™ Prestained Protein Ladder (ThermoFisher Scientific) was used as protein ladder (L).

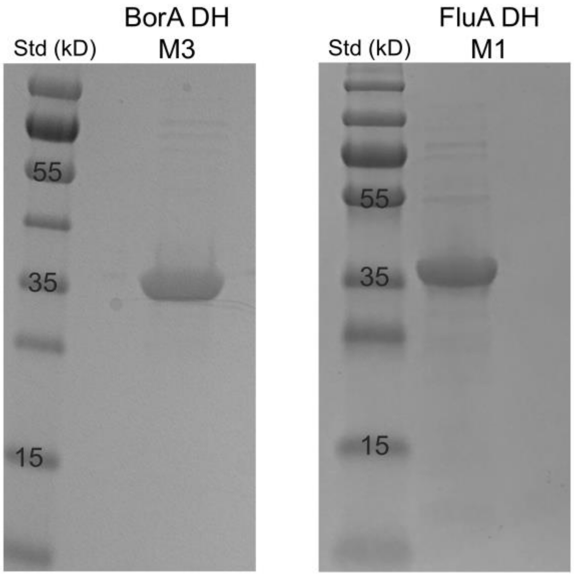

References

1. Chen VB, Arendall WB, Headd JJ, Keedy DA, Immormino RM, Kapral GJ, Murray LW, Richardson JS, Richardson DC (2010) MolProbity: all-atom structure validation for macromolecular crystallography. Acta Crystallogr D Biol Crystallogr 66:12–21. https://doi.org/10.1107/S0907444909042073
2. Engh RA, Huber R (1991) Accurate bond and angle parameters for X-ray protein structure refinement. Acta Crystallogr A Found Crystallogr 47:392–400. https://doi.org/10.1107/S0108767391001071
3. Hagen A, Poust S, Rond T de, Fortman JL, Katz L, Petzold CJ, Keasling JD (2016) Engineering a polyketide synthase for in vitro production of adipic acid. ACS Synth Biol 5:21–27. https://doi.org/10.1021/acssynbio.5b00153
4. Ham TS, Dmytriv Z, Plahar H, Chen J, Hillson NJ, Keasling JD (2012) Design, implementation and practice of JBEI-ICE: an open source biological part registry platform and tools. Nucleic Acids Res 40:e141. https://doi.org/10.1093/nar/gks531
5. Lin T-Y, Borketey LS, Prasad G, Waters SA, Schnarr NA (2013) Sequence, cloning, and analysis of the fluvirucin B1 polyketide synthase from Actinomadura vulgaris. ACS Synth Biol 2:635–642. https://doi.org/10.1021/sb4000355
